# Plasma Microrna Profiling in Overweight and Obese Pregnant Women: An Exploratory Pooled-Sample Study

**DOI:** 10.2478/bjmg-2026-0012

**Published:** 2026-10-14

**Authors:** V Spasova, D Hristova, B Rukova, L Koleva, M Vladimirov, A Kolev, D Koleva, O Antonova, B Karamishev, S Hadzhidekova, K Karamisheva

**Affiliations:** Department of Medical Genetics, Faculty of Medicine, Medical University – Sofia, Sofia, Bulgaria; Department of Immunology, National Center of Infectious and Parasitic Diseases, Sofia, Bulgaria; Clinic of Operative Gynecology, University Hospital “Pirogov”, Sofia, Bulgaria; Department of Obstetrics and Gynecology, SofiaMed University Hospital, Sofia, Bulgaria; Faculty of Medicine, Medical University – Sofia, Sofia, Bulgaria; Research Department, SBALAGRM Sofia, Sofia, Bulgaria; Second Gynecology Clinic, University Hospital “Maichin Dom”, Sofia, Bulgaria; Department of Obstetrics and Gynecology, Faculty of Medicine, Medical University – Sofia, Sofia, Bulgaria

**Keywords:** microRNA, maternal obesity, pregnancy, plasma, miR-21-5p, biomarker

## Abstract

Background: Maternal overweight and obesity are associated with metabolic, vascular, and obstetric complications. Circulating microRNAs (miRNAs) are stable in plasma and may reflect obesity-related molecular alterations during pregnancy. Aim: To compare plasma miRNA expression profiles between normal-weight and overweight/obese pregnant women. Methods: The study included 50 pregnant women: 25 with normal weight and 25 with overweight/obesity. RNA was isolated from individual plasma samples and combined into one pooled RNA sample per study group. The pooled samples were analyzed in technical triplicate using a 96-well miRCURY LNA PCR panel containing 84 target miRNA assays and quality-control/reference assays. Results: Seventeen target miRNAs met the predefined >2-fold higher-expression threshold and 15 met the <0.5-fold threshold in the over-weight/obese pool. miR-21-5p was the only target miRNA that met both the predefined twofold magnitude criterion and the nominal p < 0.05 threshold (fold change = 2.58; p = 0.030266). Conclusion: The pooled-sample analysis identifies miR-21-5p as an exploratory candidate signal associated with maternal overweight/obesity. Individual-sample testing and validation in larger independent cohorts are required before biomarker performance or clinical utility can be established.

## INTRODUCTION

Obesity is a chronic, multifactorial disease characterized by excessive adipose-tissue accumulation and metabolic dysfunction. In adults, the World Health Organization defines overweight as a body mass index (BMI) of 25.0–29.9 kg/m^2^ and obesity as a BMI of at least 30.0 kg/m^2^ [1]. During pregnancy, pre-existing overweight and obesity are associated with increased risks of gestational diabetes mellitus, hypertensive disorders, preterm birth, and delivery complications [ 2, 3, 4]. These associations have stimulated interest in circulating molecular markers that may characterize the maternal metabolic state early in pregnancy.

MicroRNAs (miRNAs) are small non-coding RNAs, typically approximately 22 nucleotides in length, that regulate gene expression post-transcriptionally [5,6]. During canonical biogenesis, a precursor hairpin generates mature strands derived from its 5′ and 3′ arms, designated -5p and -3p, respectively. These mature strands can have distinct seed sequences and therefore generally regulate different sets of messenger RNAs, although partial target overlap may occur [5]. Consistent strand-specific nomenclature is consequently important when reporting miRNA-expression findings.

MiRNAs are detectable and relatively stable in plasma and other body fluids, which supports their investigation as minimally invasive biomarkers [7]. Circulating miRNA profiles have been studied in gestational diabetes mellitus, preeclampsia, and maternal obesity [ 8, 9, 10, 11, 12, 13, 14, 15]. The study specifically recommended by the reviewer showed that plasma miR-29a was downregulated in women with gestational diabetes mellitus, whereas miR-17 and miR-181a did not differ significantly between groups [8]. These findings illustrate that statistical significance must be distinguished from non-significant directional changes.

MiR-21-5p was of particular a priori interest because it participates in inflammatory, metabolic, proliferative, and vascular signaling pathways that are relevant to both obesity and placental physiology [11,16, 17, 18]. Circulating miR-21-5p has also been reported as increased in plasma from women with preeclampsia, although its diagnostic value remains investigational [19].

The aim of this exploratory study was to compare the plasma miRNA expression profiles of normal-weight and overweight/obese pregnant women and to identify candidate miRNAs associated with maternal BMI category. Pregnancy complications and adverse pregnancy outcomes were not analyzed as study endpoints.

## MATERIALS AND METHODS

### Patients and biological samples

This exploratory study included 50 pregnant women recruited at pregnancy registration during the first trimester. The normal-weight group comprised 25 participants with BMI 18.5–24.9 kg/m², and the overweight/obese group comprised 25 participants with BMI ≥25.0 kg/m², in accordance with World Health Organization adult BMI categories [1]. Weight and height were measured at pregnancy registration, and BMI was calculated as weight in kilograms divided by height in meters squared. Inclusion criteria were ultrasound-confirmed pregnancy and available measured weight and height. Exclusion criteria were BMI <18.5 kg/m^2^, absence of ultrasound-confirmed pregnancy, diabetes, glomerulonephritis, and chronic infection.

Peripheral blood (10 mL) was collected into ethylenediaminetetraacetic acid vacuum tubes under sterile conditions and labeled with the sampling date, sampling time, and participant code. Samples were processed within 1 h of collection and centrifuged at 1,900 × g for 10 min at 4°C. The plasma phase was carefully transferred to a new tube without disturbing the buffy coat and subjected to a second centrifugation at 3,000 × g for 15 min at 4°C to remove residual cells and cellular debris. Clarified plasma was transferred to RNase-free tubes, aliquoted, and stored at −80°C until RNA extraction. Hemolysis was evaluated as part of the GeneGlobe serum/plasma quality-control analysis using the hemolysis-sensitive miRNA controls incorporated in the miRCURY LNA panel.

The study was conducted in accordance with the Declaration of Helsinki and was approved by the Ethics Committee for Scientific Research at Medical University – Sofia **[KENIMUS №11/27.05.2022]**. Written informed consent was obtained from all participants.

### RNA extraction and biobanking

Total RNA enriched for small RNAs was isolated from each participant’s plasma using the miRNeasy Serum/Plasma Advanced Kit and miRNeasy Serum/Plasma Spike-In Control (Qiagen, Hilden, Germany) according to the manufacturer’s instructions and the laboratory’s optimized protocol. The isolated RNA samples were coded and stored at −80°C. RNA quantity and purity were assessed by absorbance at 260 and 280 nm using a NanoDrop 2000c spectrophotometer (Thermo Fisher Scientific, Waltham, MA, USA), and the results were recorded in the study database.

For the expression-screening experiment, the individual RNA samples were combined into two composite samples: one pool representing the 25 normal-weight participants and one pool representing the 25 overweight/obese participants. Equal amounts of total RNA were contributed by each participant. Specifically, 50 ng RNA from each of the 25 samples was combined to generate each group-specific pool, corresponding to 1.25 µg total RNA per pool. The resulting RNA pools had concentrations of 14.85 ng/µL and 14.98 ng/µL, corresponding to approximate final volumes of 84.2 µL and 83.4 µL, respectively. A total of 150 ng pooled RNA was subsequently used for reverse transcription.

### cDNA synthesis and miRNA expression analysis

The two pooled RNA samples were reverse-transcribed using the miRCURY LNA RT Kit (Qiagen) according to the manufacturer’s instructions. Reverse transcription was performed for 60 min at 42°C followed by 5 min at 95°C for reverse-transcriptase inactivation. MiRNA expression was evaluated using the 96-well miRCURY LNA miRNA Focus PCR Panel (Qiagen; Cat. no. YAHS-206Z) and miRCURY LNA SYBR Green chemistry in a total reaction volume of 10 µL per well. The panel contained 84 target miRNA assays (positions A01–G12) together with reference/quality-control and spike-in assays. Quantitative PCR was performed on an Applied Biosystems 7500 Real-Time PCR System (Thermo Fisher Scientific) with an initial activation step at 95°C for 2 min, followed by 40 cycles of 95°C for 10 s and 56°C for 60 s, followed by melting-curve analysis. Each pooled sample was analyzed in technical triplicate. No single endogenous reference miRNA was used for normalization; GeneGlobe global-mean normalization was applied across expressed miRNA targets. Manufacturer-provided exogenous spike-in controls (UniSp2–6 and cel-miR-39-3p) were used for quality-control purposes and were not included in the global-mean normalization.

### Data and statistical analysis

Cycle-threshold (Ct) values were exported from the instrument and analyzed using the Qiagen GeneGlobe Data Analysis Center. The Ct cut-off was set at 35. Relative expression was calculated using the ΔΔCt method and fold change was reported as 2^−ΔΔCt. A twofold magnitude threshold (fold change ≥2.0 or ≤0.5) was used as an exploratory screening criterion and was not treated as evidence of statistical significance. GeneGlobe calculated p-values using a student’s t-test of replicate 2^−ΔCt values for each miRNA in the control and test groups; the software report specifies a parametric, paired, two-sample, equal-variance, two-tailed distribution. A nominal p < 0.05 threshold was used. No correction for multiple comparisons was applied. Because the replicate measurements were technical replicates of one pooled biological sample per group, the resulting p-values were interpreted as nominal and hypothesis-generating rather than as estimates of participant-level biological variability.

Accordingly, statistical results from the pooled technical replicates should not be interpreted as participant-level inference and require confirmation using individual biological samples.

## RESULTS

### Demographic and anthropometric characteristics

The study included 25 normal-weight and 25 over-weight/obese pregnant women. Demographic and anthropometric characteristics are summarized in Table 1. Mean age was 32.1 years in the normal-weight group and 31.2 years in the overweight/obese group.

**Table 1.** Demographic and anthropometric characteristics. Values are presented as mean (minimum–maximum).

**Characteristic**	**Normal-weight group (n = 25)**	**Overweight/obese group (n = 25)**
Age, years	32.1 (22–46)	31.2 (21–42)
Height, cm	166.72 (151–178)	165.78 (150–179)
BMI, kg/m^2^	21.27 (19–24)	36.31 (26–47)

### miRNA expression analysis in normal-weight and overweight/obese pregnant women

After application of the Ct cut-off, 17 of the 84 target miRNAs met the predefined >2-fold higher-expression threshold in the overweight/obese pool (Table 2,Figure 1), and 15 met the <0.5-fold threshold. Among the target miRNAs meeting the twofold magnitude criterion, miR-21-5p was the only one that also met the nominal p < 0.05 threshold (fold change = 2.58; p = 0.030266). The remaining threshold-crossing target miRNAs are described as non-significant fold-change trends rather than as statistically established up-or down-regulation. One additional target, miR-206, had a nominal p = 0.007633 but a fold change of 0.61 and therefore did not meet the predefined twofold magnitude criterion. Complete results for all 84 target miRNAs are provided in Supplementary Table S1.

**Table 2.** Target miRNAs meeting the predefined >2-fold higher-expression threshold in the overweight/obese pooled sample. P-values are nominal and unadjusted.

**miRNA**	**Fold change**	**Exact p-value**	**Interpretation**
miR-100-5p	2.67	0.293932	Non-significant fold-change trend
miR-106b-5p	2.27	0.135031	Non-significant fold-change trend
miR-133b	6.62	0.205666	Non-significant fold-change trend
miR-192-5p	2.02	0.436563	Non-significant fold-change trend
miR-200a-3p	3.97	0.353263	Non-significant fold-change trend
miR-200b-3p	3.94	0.173488	Non-significant fold-change trend
miR-20a-5p	3.66	0.314082	Non-significant fold-change trend
**miR-21-5p**	**2.58**	**0.030266**	**Meets nominal p < 0.05**
miR-214-3p	2.53	0.209446	Non-significant fold-change trend
miR-222-3p	2.45	0.138196	Non-significant fold-change trend
miR-27a-3p	5.18	0.257703	Non-significant fold-change trend
let-7c-5p	3.99	0.268329	Non-significant fold-change trend
miR-10a-5p	2.18	0.201061	Non-significant fold-change trend
miR-191-5p	2.80	0.353809	Non-significant fold-change trend
miR-22-3p	6.44	0.185505	Non-significant fold-change trend
miR-24-3p	8.49	0.344617	Non-significant fold-change trend
miR-26a-5p	3.73	0.246393	Non-significant fold-change trend

Fifteen target miRNAs met the <0.5-fold threshold in the overweight/obese pooled sample: miR-124-3p, miR-141-3p, miR-195-5p, miR-200c-3p, miR-205-5p, miR-223-3p, miR-224-5p, miR-574-3p, miR-130b-3p, miR-145-5p, miR-193a-5p, miR-30e-5p, miR-374a-5p, miR-7-5p, and miR-31-5p. None met the nominal p < 0.05 threshold. Their exact fold changes and p-values are reported in Supplementary Table S1.

**Figure 1.**
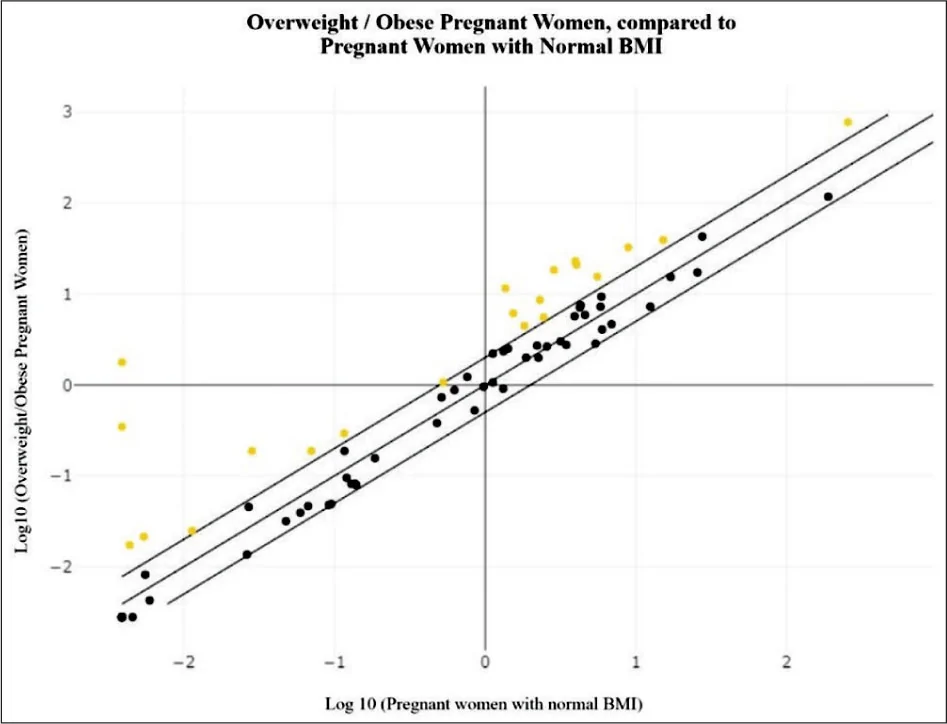
GeneGlobe scatter plot of pooled plasma miRNA-expression measurements in overweight/obese and normal-weight pregnant women. Yellow points indicate assay signals meeting the predefined twofold fold-change threshold; this visual threshold does not itself indicate statistical significance. The plot includes target and reference/quality-control miRNA assays. The central diagonal represents equal relative expression, and the outer diagonals represent twofold differences.

## DISCUSSION

In this exploratory pooled-sample study, miR-21-5p was the only target miRNA that met both the predefined twofold fold-change magnitude criterion and the nominal p < 0.05 threshold. Seventeen target miRNAs met the >2-fold threshold and 15 met the <0.5-fold threshold; these non-significant threshold-crossing findings should be interpreted as screening signals rather than as evidence of statistically established differential expression. miR-206 showed a nominal p < 0.05 without meeting the twofold magnitude criterion, further illustrating the need to distinguish fold-change magnitude from nominal statistical significance in this exploratory technical-replicate analysis.

Previous studies specifically examining circulating miRNAs in maternal obesity have reported heterogeneous expression patterns. Carreras-Badosa et al. identified 13 circulating miRNAs associated with pregestational or gestational obesity and subsequently validated selected findings in a larger cohort [12]. Enquobahrie et al. reported associations between pregnancy BMI and multiple circulating miRNAs in two pregnancy cohorts [13]. In a substantially larger first-trimester analysis, Thibeault et al. identified BMI-associated plasma miRNAs and replicated the direction and magnitude of several associations in an independent cohort [14]. Serati et al. also described distinct maternal plasma miRNA patterns in normal-weight pregnancies, obese pregnancies without gestational diabetes, and obese pregnancies with gestational diabetes [15]. Differences among studies may reflect gestational timing, study population, obesity definition, analytical platform, normalization strategy, comorbidities, and whether individual or pooled samples were analyzed.

The nominal increase in miR-21-5p is biologically plausible. MiR-21-5p is involved in inflammatory and proliferative signaling and has been investigated in metabolic disorders and pregnancy-related vascular dysfunction [11,16, 17, 18, 19]. Kolkova et al. reported a modest but statistically significant increase in plasma miR-21-5p in preeclamptic pregnancies [19]. Nevertheless, the current study did not evaluate preeclampsia, gestational diabetes, or other clinical outcomes; the present finding therefore supports only an association with BMI group in this pooled experiment and cannot establish prediction of pregnancy complications.

The practical implication of the present result is the identification of a candidate for subsequent validation rather than immediate clinical application. A confirmatory study should quantify miR-21-5p in each participant, predefine normalization and quality-control procedures, adjust for maternal age, gestational age, parity, smoking, and metabolic comorbidities, and evaluate reproducibility in an independent cohort. Prospective follow-up would then be required to determine whether miR-21-5p adds predictive value for specific pregnancy outcomes beyond established clinical risk factors.

This study has several important limitations. First, the sample size was modest. Second, the use of one pooled biological sample per group prevented assessment of interindividual variability and precluded participant-level effect estimates. Third, the findings were not independently validated using individual plasma samples or a second analytical method. Fourth, pregnancy complications and neonatal outcomes were not analyzed, so no direct conclusion can be drawn regarding prognostic performance. Fifth, 84 target miRNAs were screened and no correction for multiple comparisons was applied; all reported p-values are therefore nominal. Finally, statistical testing was based on technical replicates of pooled biological material and cannot substitute for biological replication. These limitations mean that the present signals should be considered hypothesis-generating until confirmed in adequately powered individual-sample cohorts.

## CONCLUSION

This exploratory pooled-plasma analysis identified miR-21-5p as the only target miRNA meeting both the predefined twofold magnitude criterion and the nominal p < 0.05 threshold in the overweight/obese group compared with normal-weight controls. The finding supports further investigation of miR-21-5p as a candidate marker associated with maternal overweight and obesity during pregnancy. Validation in larger independent cohorts using individual samples and prospective assessment of defined pregnancy outcomes is required before clinical utility can be established.

## Supplementary Material

Supplementary Material Details
