# Supplementary material for: Plasma Microrna Profiling in Overweight and Obese Pregnant Women: An Exploratory Pooled-Sample Study
Source: Balkan J Med Genet. 2026 Oct 14;29(1):1–6. doi: 10.2478/bjmg-2026-0012 (PMC13667939; doi:10.2478/bjmg-2026-0012)
Supplement: Supplementary file 1 [file bjmg-2026-0012-supplementary_materials.pdf]

BJMG 29 (1), 2026 • 1-3
DOI: 10.2478/bjmg-2026-0012

PLASMA MICRORNA PROFILING IN OVERWEIGHT AND OBESE PREGNANT WOMEN: AN EXPLORATORY POOLED-SAMPLE STUDY

Spasova V[1], Hristova D[2], Rukova B[1], Koleva L[3,4], Vladimirov M[5,6]*, Kolev A[5], Koleva D[5], Antonova O[1], Karamishev B[5], Hadzhidekova S[1], Karamisheva K[7,8]

*Corresponding Author: Martin Vladimirov, SBALAGRM Sofia, 5B Baku Str., 1700 Sofia, Bulgaria; email: mvladimirov11@gmail.com; telephone: +359 889 59 1101

Supplementary Table S1. Complete GeneGlobe results for the 84 target miRNA assays

Position	miRNA	Fold change	Exact p-value	Ct normal-weight pool	Ct overweight/obese pool	Twofold magnitude threshold
A01	let-7a-5p	1.61	0.881874	27.39	26.22	No
A02	miR-1-3p	0.70	0.418381	30.90	30.94	No
A03	miR-100-5p	2.67	0.293932	30.83	28.93	Yes
A04	miR-106b-5p	2.27	0.135031	25.71	24.05	Yes
A05	miR-10b-5p	1.42	0.863422	27.96	26.97	No
A06	miR-122-5p	0.84	0.448396	29.42	29.20	No
A07	miR-124-3p	0.47	0.264783	33.76	34.35	Yes
A08	miR-125b-5p	0.79	0.442878	28.06	27.91	No
A09	miR-126-3p	1.45	0.482357	25.02	24.01	No
A10	miR-133a-3p	1.61	0.739400	30.10	28.93	No
A11	miR-133b	6.62	0.205666	32.14	28.93	Yes
A12	miR-134-5p	0.60	0.314949	29.86	30.12	No
B01	miR-141-3p	0.39	0.091922	29.83	30.71	Yes
B02	miR-143-3p	1.77	0.580308	26.59	25.29	No
B03	miR-146a-5p	0.69	0.943292	26.60	26.66	No
B04	miR-150-5p	0.53	0.157196	24.57	25.01	No
B05	miR-155-5p	0.79	0.550608	30.05	29.91	No
B06	miR-17-5p	0.68	0.869573	24.42	24.49	No
B07	miR-17-3p	0.66	0.469770	31.07	31.19	No
B08	miR-18a-5p	1.96	0.262347	26.83	25.38	No
B09	miR-192-5p	2.02	0.436563	27.92	26.43	Yes
B10	miR-195-5p	0.47	0.426895	30.43	31.03	Yes
B11	miR-196a-5p	0.72	0.179957	35.00	35.00	No
B12	miR-19a-3p	0.67	0.114895	22.32	22.41	No
C01	miR-19b-3p	0.90	0.744957	22.91	22.58	No
C02	miR-200a-3p	3.97	0.353263	34.83	32.36	Yes
C03	miR-200b-3p	3.94	0.173488	34.52	32.06	Yes
C04	miR-200c-3p	0.30	0.246509	29.69	30.97	Yes
C05	miR-203a-3p	0.72	0.532682	34.39	34.39	No
C06	miR-205-5p	0.32	0.489079	29.80	30.97	Yes
C07	miR-208a-3p	0.72	0.179957	35.00	35.00	No

Continues on the next page

Continuation of the previous page

Position	miRNA	Fold change	Exact p-value	Ct normal-weight pool	Ct overweight/obese pool	Twofold magnitude threshold
C08	miR-20a-5p	3.66	0.314082	23.84	21.49	Yes
**C09**	**miR-21-5p**	**2.58**	**0.030266**	**23.07**	**21.22**	**Yes**
C10	miR-210-3p	1.40	0.598077	27.67	26.71	No
C11	miR-214-3p	2.53	0.209446	30.10	28.29	Yes
C12	miR-215-5p	0.61	0.515571	27.23	27.45	No
D01	miR-221-3p	1.22	0.364723	25.85	25.08	No
D02	miR-222-3p	2.45	0.138196	26.13	24.36	Yes
D03	miR-223-3p	0.33	0.125770	20.33	21.47	Yes
D04	miR-224-5p	0.38	0.276757	29.78	30.68	Yes
D05	miR-23a-3p	0.68	0.782313	24.21	24.30	No
D06	miR-25-3p	0.58	0.294273	23.36	23.66	No
D07	miR-27a-3p	5.18	0.257703	24.98	22.13	Yes
D08	miR-296-5p	0.52	0.226884	30.44	30.90	No
D09	miR-29a-3p	0.95	0.828958	26.82	26.43	No
D10	miR-30d-5p	0.80	0.660295	25.21	25.05	No
D11	miR-34a-5p	0.52	0.401219	32.24	32.71	No
D12	miR-375-3p	1.69	0.424777	32.21	30.97	No
E01	miR-423-5p	0.98	0.725752	27.03	26.59	No
E02	miR-499a-5p	0.72	0.179957	35.00	35.00	No
E03	miR-574-3p	0.32	0.421542	29.90	31.07	Yes
E04	miR-885-5p	0.57	0.113179	29.83	30.18	No
E05	miR-9-5p	1.47	0.343183	34.49	33.45	No
E06	miR-92a-3p	1.55	0.328061	22.21	21.10	No
E07	miR-93-5p	1.24	0.447584	24.45	23.66	No
E08	let-7c-5p	3.99	0.268329	26.38	23.90	Yes
E09	miR-107	1.74	0.565905	24.89	23.61	No
E10	miR-10a-5p	2.18	0.201061	33.45	31.85	Yes
E11	miR-128-3p	0.63	0.587514	29.95	30.13	No
E12	miR-130b-3p	0.25	0.267177	27.86	29.41	Yes
F01	miR-145-5p	0.40	0.192250	24.61	25.45	Yes
F02	miR-148a-3p	1.28	0.907662	24.80	23.96	No
F03	miR-15a-5p	1.68	0.313384	24.91	23.69	No
F04	miR-184	0.72	0.179957	35.00	35.00	No
F05	miR-193a-5p	0.13	0.172959	28.30	30.75	Yes
F06	miR-204-5p	0.66	0.240595	31.38	31.49	No
**F07**	**miR-206**	**0.61**	**0.007633**	**34.77**	**35.00**	**No**
F08	miR-211-5p	0.72	0.179957	35.00	35.00	No
F09	miR-26b-5p	0.88	0.789872	25.82	25.52	No
F10	miR-30e-5p	0.42	0.324282	24.45	25.24	Yes
F11	miR-372-3p	0.72	0.179957	35.00	35.00	No
F12	miR-373-3p	0.72	0.179957	35.00	35.00	No
G01	miR-374a-5p	0.46	0.342453	24.65	25.28	Yes
G02	miR-376c-3p	1.03	0.917438	25.63	25.11	No
G03	miR-7-5p	0.28	0.191847	29.20	30.56	Yes
G04	miR-96-5p	0.52	0.323422	30.39	30.87	No

Continues on the next page

Continuation of the previous page

Position	miRNA	Fold change	Exact p-value	Ct normal-weight pool	Ct overweight/obese pool	Twofold magnitude threshold
G05	miR-103a-3p	0.95	0.978441	25.33	24.93	No
G06	miR-15b-5p	1.07	0.905168	26.09	25.52	No
G07	miR-16-5p	0.62	0.217166	19.44	19.64	No
G08	miR-191-5p	2.80	0.353809	24.52	22.56	Yes
G09	miR-22-3p	6.44	0.185505	25.48	22.31	Yes
G10	miR-24-3p	8.49	0.344617	26.55	22.99	Yes
G11	miR-26a-5p	3.73	0.246393	25.79	23.41	Yes
G12	miR-31-5p	0.36	0.311239	32.09	33.09	Yes

Notes: The table contains the 84 target assays at positions A01–G12 in the GeneGlobe report. Row-H reference/quality-control and spike-in assays are not included. Fold change was calculated by GeneGlobe using the $2^{-\Delta\Delta Ct}$ method after global-mean normalization. The Ct cut-off was 35. The "twofold magnitude threshold" column indicates fold change ≥2.0 or ≤0.5 and is an exploratory screening criterion, not a test of statistical significance. P-values are nominal GeneGlobe Student's t-test results from technical replicates; no multiple-testing correction was applied. miR-21-5p (fold change 2.58; $p = 0.030266$) met both the twofold magnitude and nominal $p < 0.05$ thresholds. miR-206 had $p = 0.007633$ but did not meet the twofold magnitude threshold (fold change 0.61).

Abbreviations: Ct, cycle threshold; miRNA, microRNA.
